# Community-level chlamydial serology for assessing trachoma elimination in trachoma-endemic Niger

**DOI:** 10.1371/journal.pntd.0007127

**Published:** 2019-01-28

**Authors:** Jessica S. Kim, Catherine E. Oldenburg, Gretchen Cooley, Abdou Amza, Boubacar Kadri, Baido Nassirou, Sun Yu Cotter, Nicole E. Stoller, Sheila K. West, Robin L. Bailey, Jeremy D. Keenan, Bruce D. Gaynor, Travis C. Porco, Thomas M. Lietman, Diana L. Martin

**Affiliations:** 1 F. I. Proctor Foundation, University of California San Francisco, San Francisco, California, United States of America; 2 Department of Ophthalmology, University of California San Francisco, San Francisco, California, United States of America; 3 Department of Epidemiology and Biostatistics, University of California San Francisco, San Francisco, California, United States of America; 4 Divison of Parasitic Diseases and Malaria, Centers for Disease Prevention and Control, Atlanta, Georgia, United States of America; 5 Programme FSS/Université Abdou Moumouni de Niamey, Programme Nationale des Soins Oculaire, Niger; 6 Dana Center for Preventive Ophthalmology, Wilmer Eye Institute, Johns Hopkins University, Baltimore, Maryland, United States of America; 7 Clinical Research Unit, Department of Infectious and Tropical Diseases, London School of Hygiene and Tropical Medicine, London, United Kingdom; 8 Institute for Global Health, University of California San Francisco, San Francisco, California, United States of America; Mohammed Bin Rashid University of Medicine and Health Sciences, UNITED ARAB EMIRATES

## Abstract

**Background:**

Program decision-making for trachoma elimination currently relies on conjunctival clinical signs. Antibody tests may provide additional information on the epidemiology of trachoma, particularly in regions where it is disappearing or elimination targets have been met.

**Methods:**

A cluster-randomized trial of mass azithromycin distribution strategies for trachoma elimination was conducted over three years in a mesoendemic region of Niger. Dried blood spots were collected from a random sample of children aged 1–5 years in each of 24 study communities at 36 months after initiation of the intervention. A multiplex bead assay was used to test for antibodies to two *Chlamydia trachomatis* antigens, Pgp3 and CT694. We compared seropositivity to either antigen to clinical signs of active trachoma (trachomatous inflammation—follicular [TF] and trachomatous inflammation—intense [TI]) at the individual and cluster level, and to ocular chlamydia prevalence at the community level.

**Results:**

Of 988 children with antibody data, TF prevalence was 7.8% (95% CI 6.1 to 9.5) and TI prevalence was 1.6% (95% CI 0.9 to 2.6). The overall prevalence of antibody positivity to Pgp3 was 27.2% (95% CI 24.5 to 30), and to CT694 was 23.7% (95% CI 21 to 26.2). Ocular chlamydia infection prevalence was 5.2% (95% CI 2.8 to 7.6). Seropositivity to Pgp3 and/or CT694 was significantly associated with TF at the individual and community level and with ocular chlamydia infection and TI at the community level. Older children were more likely to be seropositive than younger children.

**Conclusion:**

Seropositivity to Pgp3 and CT694 correlates with clinical signs and ocular chlamydia infection in a mesoendemic region of Niger.

**Trial registration:**

ClinicalTrials.gov NCT00792922.

## Author summary

Trachoma programs currently use the clinical sign of trachomatous inflammation-follicular (TF) to guide community treatment decisions and evaluate response to mass drug administration with azithromycin. These programs rely on clinical grading that poorly correlates with infection with the causative agent of trachoma, *Chlamydia trachomatis (Ct)*, in low prevalence areas. Serologic measures of *Ct* may provide additional information about exposure and transmission patterns. Here, we evaluated the relationship between serologic markers of *Ct*, infection, and TF at the individual and community levels to evaluate the utility of serology for measuring trachoma in a mesoendemic region of Niger. We found that serologic markers correlated with both infection and TF, indicating that inclusion of serologic markers may be useful to guide trachoma decision making.

## Introduction

Trachoma, caused by repeated ocular infection with *Chlamydia trachomatis* (*Ct*), has been targeted by the World Health Organization (WHO) for elimination as a public health problem by the year 2020. As part of the strategy to achieve elimination, WHO recommends annual mass drug administration (MDA) of azithromycin in endemic districts [1]. Program targets related to MDA focus on the district-level prevalence of trachomatous inflammation—follicular (TF) amongst children aged 1–9 years. To monitor progress towards elimination, population-based impact surveys are recommended to evaluate whether a district has reached the threshold of less than 5% TF prevalence in 1–9-year-olds and can cease azithromycin distribution. Two years after cessation of MDA, a surveillance survey to ensure that district-wide TF prevalence in 1–9-year-olds remains below 5% is conducted prior to the validation process. Currently, there are no guidelines for post-validation surveillance.

These surveys rely on a clinical grading scheme that is relatively inexpensive and simple to perform, but is poorly correlated with ocular *Ct* infection in low-prevalence settings [2]. Following MDA, the clinical sign trachomatous inflammation—intense (TI) has been shown to correlate better with infection than TF does [3]. However the measurement of clinical signs is subject to inter-grader variability and lack of real-time auditing since grading is performed in the field and thus can only later be validated or audited if images are taken. As trachoma elimination programs stand to benefit from an accurate, reproducible assessment of trachoma prevalence, other testing methods may be useful to help guide program decisions. These include tests of infection (polymerase chain reaction [PCR] testing of ocular swabs) and antibody-based testing [4–7].

Antibodies to *Ct* antigens may act as markers of cumulative exposure to *Ct*. Two previously described *Ct* antigens, Pgp3 and CT694, have been shown to be reactive against sera in young children living in trachoma-endemic communities [4,7,8]. At the individual level, antibodies to these proteins demonstrate high sensitivity to ocular infection and high specificity against non-endemic control specimens [8–10]. However, individual associations may not always hold at the community level, and trachoma elimination programs treat ocular *Ct* infection on a population level. Additionally, as antibody markers are not yet widely used to assess for *Ct* prevalence, better characterization of how seropositivity compares to other methods of assessing trachoma prevalence is necessary. Here, we evaluate the association between seropositivity, PCR positivity, and clinical signs of active trachoma (TF and TI) at the individual and community level in a region of Niger where some trachoma transmission is occurring (TF prevalence approximately 25% at baseline). Data were collected during the final follow-up visit of the Partnership for the Rapid Elimination of Trachoma (PRET)-Niger trial, in which communities were randomized to receive annual or biannual oral azithromycin for 3 years in order to assess the impact of treatment frequency on ocular chlamydia infection [11].

## Methods

### Study design

The study methods have been previously reported in detail elsewhere [11–13]. Briefly, a cluster randomized trial of annual versus biannual mass azithromycin distribution for trachoma control was conducted in the Matameye district of the Zinder region of Niger from May 2010 until August 2013 [4–6]. Data on active trachoma and ocular infection were collected biannually on children aged 0–5 years; dried blood spots for serological analysis were collected only at the 36-month time point and only from children aged 1–5 years. Dried blood spots were shipped to CDC at ambient temperature and tested for antibodies from July to August 2014.

### Site selection

Communities were chosen from among six different catchment areas for primary health care facilities and were eligible for inclusion if they met the following criteria: (1) contained a population between 250 to 600 persons, (2) were located more than 4 kilometers from the center of any semi-urban area, and (3) had a prevalence of active trachoma more than 10% in children aged 0–5 years [11]. 235 communities in the 6 health centers were deemed eligible, of which 48 were randomly selected for inclusion in the trial. Children aged 1–5 years were included in this analysis, due to the inability of antibody tests to differentiate between maternal-child antibodies in <1–year-olds.

### Community randomization

48 communities were randomly divided into 4 treatment arms in a 2x2 factorial design (12 communities per arm), comparing two azithromycin coverage targets (standard versus enhanced coverage) and annual versus biannual treatment. Randomization of communities to treatment arms was done using RANDOM and SORT functions in Microsoft Excel (Version 2003). Only communities from the enhanced coverage arms were included in testing for antibodies (N = 24 communities) for logistical reasons.

### Census

Trained study health workers conducted a full household census in all communities prior to the initial survey visit. During the baseline visit, adults in the household consented to census data collection, and study personnel recorded the name, sex, and age or date of birth for all individuals in the household.

### Trachoma grading and conjunctival swab collection

After consent was obtained, study participants were examined for the presence of TF and TI. Clinical grading of each everted superior tarsal conjunctiva was performed using a 2.5x binocular loupe and a torch light, if necessary, per the WHO grading system. Clinical grading was performed according to the WHO simplified grading system of TF being the presence 5 or more follicles >0.5 mm in diameter and TI as inflammation severe enough to obscure 50% of deep tarsal vessels in one or both eyes [14]. Prior to swabbing, a trained photographer took at least 2 photographs of the right eyelid of all participants using a Nikon D-series camera and a Micro Nikon 105 mm; f/2.8 lens (Nikon, Tokyo, Japan). After conjunctival examination, a Dacron swab was passed 3 times over the right upper tarsal conjunctiva, rotating the swab approximately 120 degrees between each pass. All samples were placed immediately on cold packs in the field and transferred to -20°C within 10 hours, then shipped on cold packs to University of California, San Francisco, CA, USA where they were stored in -80°C freezers until processing.

### PCR testing

PCR testing was performed for children aged 0–5 years. Samples from the same village, age in years, and visit were randomly pooled into groups of five for group testing, with a possible remainder pool of one to four samples [15]. Pooled samples were tested for the presence of *Ct* DNA using the Roche Amplicor qualitative PCR assay (Roche Molecular Systems, Indianapolis, IN, USA). Community prevalence was estimated from the pools as previously described [11,15].

### Interventions

In communities randomized to annual treatment, study participants age 6 months of age and older received a directly observed dose of oral azithromycin (20 mg/kg up to a maximum dose of 1 g in adults). In biannually treated communities, only children up to 12 years of age were offered treatment. Children under 6 months of age in all communities were offered topical tetracycline ointment (1%) to be applied to both eyes twice a day for six weeks. Pregnant women in the annual arm and individuals allergic to macrolides were offered topical tetracycline. All communities were visited up to four days in order to achieve 90% treatment coverage [12,13].

### Blood collection and antibody testing

Children under 5 years of age were selected randomly in each village for blood sample collection via finger stick or heel stick, with a goal of 50 children per village. Blood spots were analyzed for antibody to *Ct* antigens Pgp3 and CT694 using a multiplex bead array assay on a Luminex 200 platform, as previously described [7]. Results were reported as median fluorescence intensity minus background (MFI-BG) where background is the signal from beads run with buffer only. Positivity cut-off for Pgp3 was greater than or equal to 1083, and CT694 cutoff was greater than or equal to 496 as determined by receiver operator characteristic (ROC) curve analysis from a pediatric U.S.-based negative panel (N = 117) and Tanzania based positive panel from children with ocular Ct infection (N = 40) [7].

### Statistical analysis

Data were entered into a customized database (Microsoft Access v2007) developed at the Dana Center, Johns Hopkins University. To estimate associations between seropositivity, clinical trachoma, and age at the individual level, we used generalized linear models with a binomial distribution and log link to estimate prevalence ratios (PR). All standard errors were clustered at the community level, which was the randomization unit of the study. As individual-level PCR data were not available, associations between seropositivity to the *Ct* antigen and ocular chlamydia infection were conducted only at the community level. We additionally analyzed the association between seropositivity and clinical trachoma at the cluster level. We used linear regression models to evaluate relationships between trachoma indicators at the community level. All analyses were conducted in Stata 14.1 (StataCorp, College Station, TX).

### Ethics

All procedures and protocols for this study were approved by the Committee for Human Research of UCSF, and le Comité Consultatif National d’Ethique du Minstère de la Santé Publique, Niger (Ethical Committee, Niger Ministry of Health). The study’s Data Safety and Monitoring Committee observed the study implementation during annual reviews of quality assurance, as appointed by the PRET study Executive Committee. All village leaders of communities within the study agreed to participate in the trial with written (thumbprint) consent. For children under the age of 16, consent was given by a parent or a guardian. All persons participating in the trial were given the opportunity to be treated according to their community’s random treatment assignment. Communities not included in the study were offered treatment through the national treatment program. CDC personnel did not have access to personal identifying information and were determined to be non-engaged in the study.

## Results

At the 36-month follow-up, 988 1–5-year-old children from 24 communities had clinical trachoma and serology data available. TF prevalence was 7.8% (95% CI 6.1 to 9.5%) and TI prevalence was 1.6% (95% CI 0.9 to 2.6%) (Table 1). The overall prevalence of antibody positivity to Pgp3 was 27.2% (95% CI 24.5 to 30%), and to CT694 was 23.7% (95% CI 21 to 26.2%). Community prevalence of ocular chlamydia in 0-5-year-olds was 5.2% (95% CI 2.8 to 7.6%).

**Table 1:** Community and individual-level trachoma indicators, overall and by age group.

	N	TF+	TI+	Pgp3+	CT694+	PCR+^1^
Community-level prevalence, (95% CI)	24	7.1% (4.2 to 10.0%)	1.8% (0.4 to 3.2%)	25.6% (17.4 to 33.8)	22.3% (16.7 to 27.9%)	5.2% (2.8 to 7.6%)
Individual-level prevalence, N (%)	988	77 (7.8%)	16 (1.6%)	269 (27.2%)	234 (23.7%)	
Age Subgroup (year) Individual-level, N, (%)						
1	212	12 (5.7%)	5 (2.4%)	31 (14.6%)	32 (15.1%)	
2	191	18 (9.4%)	3 (1.6%)	37 (19.4%)	32 (16.8%)	
3	173	14 (8.1%)	3 (1.7%)	41 (23.7%)	42 (24.3%)	
4	197	19 (9.6%)	3 (1.5%)	67 (34.0%)	49 (24.9%)	
5	215	14 (6.5%)	2 (0.9%)	93 (43.3%)	79 (36.7%)	
Community-level association with Pgp3/CT694^2^, mean difference (95% CI)	24	0.25 (0.13 to 0.36)	0.07 (0.004 to 0.14)			0.19 (0.08 to 0.29)
Individual-level association with Pgp3/CT694^3^, prevalence ratio (95% CI)	988	1.90 (1.49 to 2.43)	1.65 (0.99 to 2.75)			

Abbreviations: TF, trachomatous inflammation—follicular; TI, trachomatous inflammation—intense; CI, confidence interval

^1^Due to pooling, only community-level prevalence estimates are available for *C*. *trachomatis* infection as determined by PCR

^2^Linear regression model

^3^Generalized linear model with a binomial distribution and log link, with standard errors clustered at the community level

### Individual-level association of seropositivity with active trachoma

Children with antibodies to either Pgp3 or CT694 were more likely to have TF (PR, 1.90, 95% CI 1.49 to 2.43, *P*<0.001) and also TI, although the latter relationship was not statistically significant (PR 1.65, 95% CI 0.99 to 2.75, *P* = 0.06).

### Community-level association of seropositivity with active trachoma and ocular chlamydia infection

**Fig 1:**
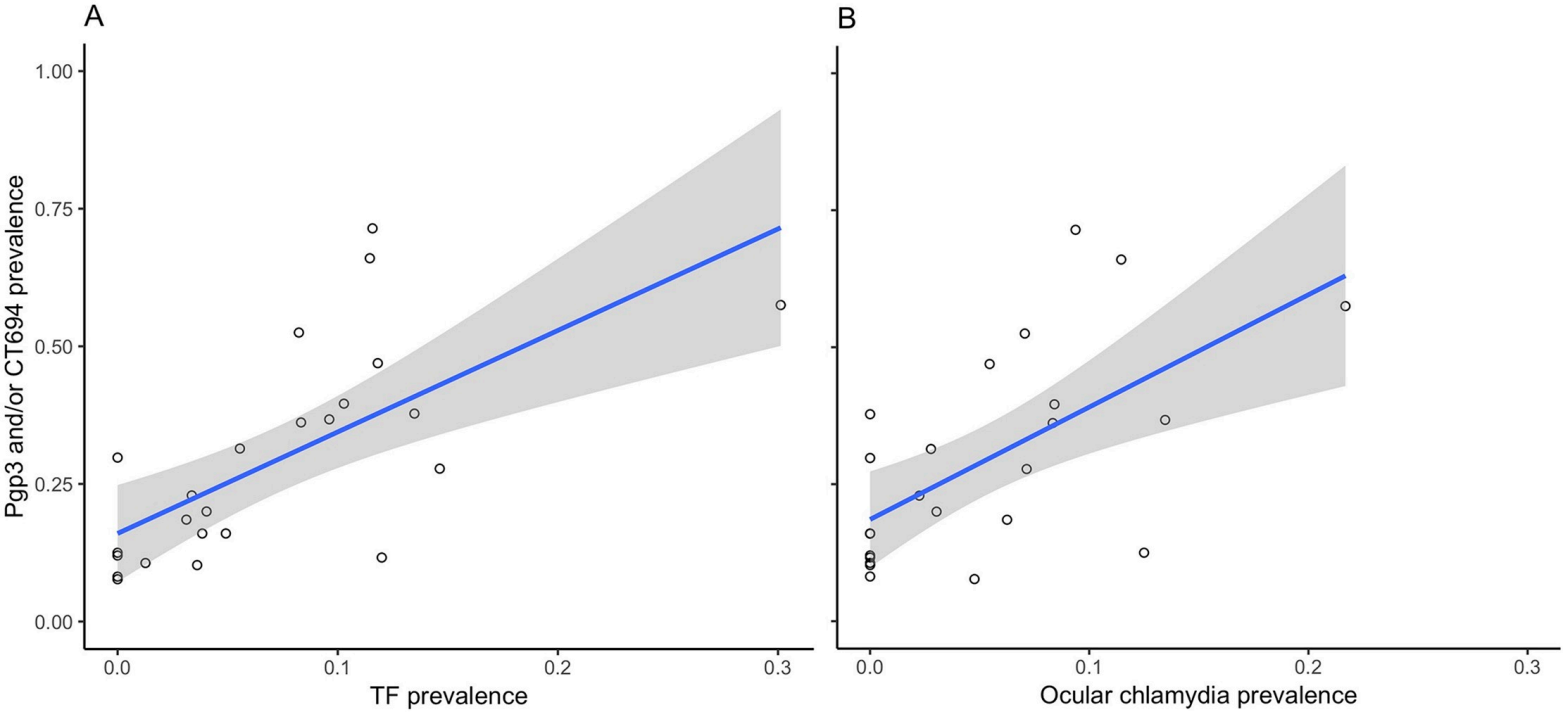
Community level association between seropositivity to Pgp3 and/or CT694 and trachoma indicators. Fig 1A depicts the association between seropositivity to Pgp3 and/or CT694 and trachomatous inflammation–follicular (TF), with community-level seroprevalence on the Y-axis and community-level TF prevalence on the X-axis. Fig 1B depicts the association between seropositivity to Pgp3 and/or CT694 and ocular chlamydia infection, with community-level seroprevalence on the Y-axis and community-level ocular chlamydia prevalence on the X-axis. Circles represent individual communities, blue lines represent the linear regression line and grey shading represents 95% confidence intervals.

Fig 1 shows the community-level prevalence of TF and ocular chlamydia compared to seropositivity to Pgp3 and/or CT694. At the community level, Pgp3 and/or CT694 seroprevalence was significantly correlated with ocular chlamydia infection (linear regression coefficient 0.19, 95% CI 0.08 to 0.29, *P* = 0.001), TF prevalence (linear regression coefficient 0.25, 95% CI 0.13 to 0.36, *P*<0.001), and TI prevalence (linear regression coefficient 0.07, 95% CI 0.004 to 0.14, *P* = 0.04).

### Age trends

**Fig 2:**
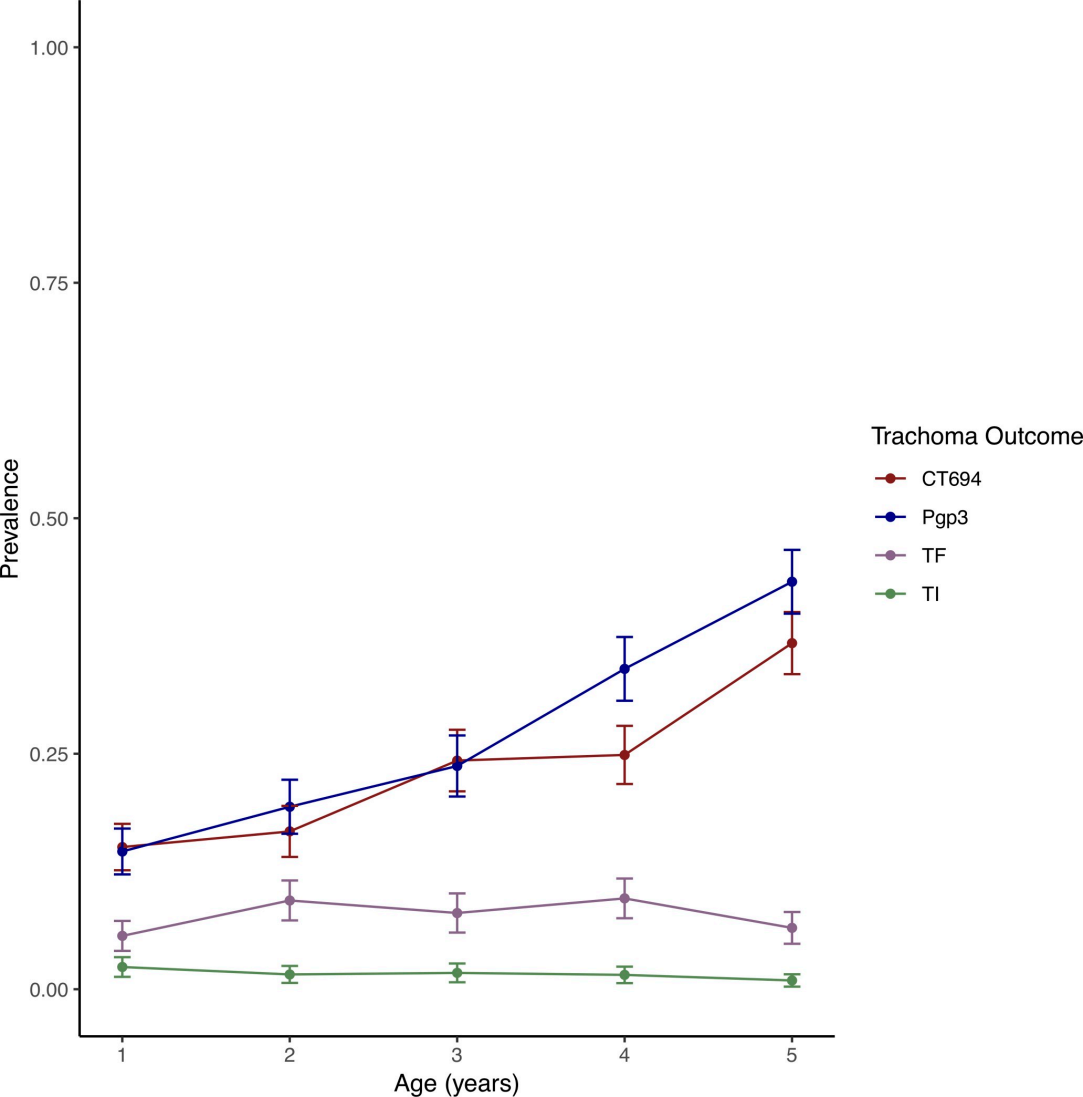
Prevalence of antibodies recognizing Pgp3 (blue) and CT694 (red), trachomatous inflammation—follicular (TF, purple) and trachomatous inflammation—intense (TI, green) by age in years.

The probability of having an antibody response to Pgp3 or CT694 increased with increasing age (PR 1.25 per one-year increase in age, 95% CI 1.17 to 1.35, *P-*trend<0.001; Fig 2). Older age was not significantly associated with a diagnosis of TF (PR 1.02 per year, 95% CI 0.89 to 1.16, *P*-trend = 0.82) or TI (PR 0.87, 95% CI 0.60 to 1.25, *P-*trend = 0.45).

### Annual vs biannual azithromycin distribution

There was no significant difference between study arms in the percentage of children antibody-positive to Pgp3 (PR 0.72 biannual versus annual, 95% CI 0.39 to 1.33, *P* = 0.29) or CT694 (PR 0.81 biannual versus annual, 95% CI 0.51 to 1.28, *P* = 0.36; Table 2).

**Table 2:** Community-level trachoma indicators by study arm.

	Annual (N = 12)	Biannual (N = 12)
TF prevalence (95% CI)	8.9% (3.3 to 14.4%)	5.4% (2.9 to 7.9%)
TI prevalence (95% CI)	1.7% (0 to 3.9%)	1.9% (0 to 4.0%)
*C*. *trachomatis* prevalence (95% CI)	7.1% (2.7 to 11.4%)	3.3% (1.0 to 5.5%)
Seropositivity to Pgp3 (95% CI)	28.4% (16.5 to 40.3%)	22.8% (9.8 to 35.8%)
Seropositivity to CT694 (95% CI)	23.5% (14.3 to 32.6%)	21.0% (13.1 to 29.0%)

## Discussion

The presence of antibodies to *Ct* antigens was correlated with both TF and ocular *Ct* infection following a 36-month annual or biannual mass azithromycin distribution program in a trachoma-endemic region of Niger. However, there was no difference in serologic outcomes by study arm, consistent with clinical data suggesting that biannual treatment did not significantly alter transmission of ocular chlamydia compared to annual treatment [11]. That serologic outcomes were consistent with other trachoma indicators supports the finding that antibodies to *Ct* antigens are correlated with TF and ocular *Ct* infection and provide complementary information. Future work evaluating serologic outcomes in a trial with a significant effect on ocular *Ct* infection or TF would provide additional evidence about the relationship between these indicators.

Currently, decision-making for MDA in trachoma elimination programs relies solely on clinical grading of TF. Grading of clinical trachoma is subjective, and prevalence surveys have demonstrated that there is poor agreement between clinical disease and ocular *Ct* infection, particularly after multiple rounds of antibiotic treatment [2,3,14,16]. Additionally, TF may be observed in the absence of infection, either as residual inflammation from the etiologic agent *Ct*[17,18], or due to other non-chlamydial bacteria [19]. Point prevalence of TF, or a test of infection, reflects disease or infection state at a current point in time, whereas serologic patterns may allow for identification of longer-term patterns in *Ct* transmission [7,8]. Anti-*Ct* antibody responses increased with age in this study, whereas TF prevalence was not significantly different across age groups, suggesting that antibody positivity rates represent the pool of exposed individuals rather than currently or recently-infected ones.

While PCR assessment of ocular *Ct* infection and clinical grading for TF and TI represent cross-sectional prevalence of trachoma, age-seroprevalence curves may provide additional insight to changes in transmission of ocular *Ct* over time. PCR or NAAT testing has historically been too costly for use in program settings[20] but cost-effective PCR tests are now being evaluated in program contexts [6]. In ocular swab specimens, PCR tests for *Ct* infection are a more specific indicator for the causative agent of trachoma than antibody testing in sera, as antibody responses to the antigens we studied cannot differentiate between exposure to ocular *Ct* and other chlamydial infections. Perinatal transmission of *Ct* from mothers with urogenital chlamydia could potentially lead to seropositivity among young children, as could sexual exposure in individuals after the age of sexual debut. However, focusing on the younger children and on the age-seroprevalence curve rather than absolute rates of seropositivity may allow for distinction between ongoing ocular *Ct* transmission and a single exposure. For example, in the Solomon Islands, a lack of increase in seropositivity to Pgp3 in 1–9-year-olds correlated with low infection rates, despite the 26% prevalence of TF, contrasting to the steep increase in seropositivity with age observed amongst 1–9-year-olds in Kiribati where TF prevalence was 28% and infection prevalence was 24% [21,22].

Antibody testing for chlamydial antigens has been conducted in a number of trachoma program settings. In treatment-naïve communities, the slope of the age seroprevalence curve increased with increasing community TF prevalence [4,21]. A significant decline in antibody responses has been shown following mass azithromycin distribution compared to pre-treatment levels in a cross-sectional study [8]. In this mesoendemic region of Niger, we noted more than 30% prevalence of antibodies to Pgp3 and CT694, consistent with results from mesoendemic communities in Tanzania [4]. In settings where surveillance surveys for trachoma elimination have been conducted, age seroprevalence curves corresponded to decreases in trachoma transmission [23]. Seroprevalence of antibodies to Pgp3 in children ages 1 to 9 years in these surveys ranged from less than 2% in some surveys[24,25] to as high as 7.5%[26] but without a steep increase in age seroprevalence curves seen in settings with ongoing transmission. The current data add to this body of knowledge by evaluating antibody responses at impact surveys after 3 rounds of MDA, a program setting for which limited data exist.

TF prevalence in this study was 7.5% after 3 years of MDA and thus antibody data do not come from a setting in which elimination thresholds have been achieved. Furthermore, the communities included in this analysis were treated with an enhanced coverage target, with up to four days of treatment. Antibiotic coverage may have been higher than is seen in programmatic or trial settings with lower coverage targets. The antibody responses and curves therefore may not be representative of what would be seen in a previously-endemic setting that has reached the elimination threshold. Trachoma programs typically include children up to age 9 in monitoring. Other studies have shown further increases in antibody responses in children aged 6–9 years[21,26], and inclusion of those ages here may have improved our ability to draw inferences from the shape of the curve. Since ocular swabs were pooled for PCR analysis, we were unable to obtain individual-level correlations between PCR and antibody positivity.

WHO currently recommends use of TF for deciding programmatic endpoints. Laboratory testing, including *Ct* serology, could be used as a supplement or replacement for TF when conducting surveillance after validation of the elimination of trachoma as a public health problem, given serology is generally inexpensive, objective, and provides estimates of exposure over time.[26] The elimination thresholds do not require a complete absence of ocular *Ct* infection, and therefore infection may still be present in communities that have reached the district-wide elimination threshold, as was seen in Tanzania where infection was present but did not lead to re-emergence.[27] Having a test of exposure, or of repeated infection, would allow more complete evaluation of the history of exposure to ocular *Ct* in children.

The findings and conclusions in this report are those of the authors and do not necessarily represent the official position of the Centers for Disease Control and Prevention.

## Supporting information

S1 Checklist STROBE checklist for cross-sectional studies. (DOC)
